# A deep learning methodology for the automated detection of end-diastolic frames in intravascular ultrasound images

**DOI:** 10.1007/s10554-021-02162-x

**Published:** 2021-02-15

**Authors:** Retesh Bajaj, Xingru Huang, Yakup Kilic, Ajay Jain, Anantharaman Ramasamy, Ryo Torii, James Moon, Tat Koh, Tom Crake, Maurizio K. Parker, Vincenzo Tufaro, Patrick W. Serruys, Francesca Pugliese, Anthony Mathur, Andreas Baumbach, Jouke Dijkstra, Qianni Zhang, Christos V. Bourantas

**Affiliations:** 1 Department of Cardiology, Barts Heart Centre, Barts Health NHS Trust, West Smithfield, London, EC1A 7BE UK; 2 Centre for Cardiovascular Medicine and Devices, William Harvey Research Institute, Queen Mary University of London, London, UK; 3 School of Electronic Engineering and Computer Science, Queen Mary University of London, London, UK; 4 Department of Mechanical Engineering, University College London, London, UK; 5 Institute of Cardiovascular Sciences, University College London, London, UK; 6 Faculty of Medicine, National Heart & Lung Institute, Imperial College London, London, UK; 7 Department of Radiology, Division of Image Processing, Leiden University Medical Center, Leiden, The Netherlands

**Keywords:** Intravascular ultrasound, Near-infrared spectroscopy, Machine learning

## Abstract

Coronary luminal dimensions change during the cardiac cycle. However, contemporary volumetric intravascular ultrasound (IVUS) analysis is performed in non-gated images as existing methods to acquire gated or to retrospectively gate IVUS images have failed to dominate in research. We developed a novel deep learning (DL)-methodology for end-diastolic frame detection in IVUS and compared its efficacy against expert analysts and a previously established methodology using electrocardiographic (ECG)-estimations as reference standard. Near-infrared spectroscopy-IVUS (NIRS-IVUS) data were prospectively acquired from 20 coronary arteries and co-registered with the concurrent ECG-signal to identify end-diastolic frames. A DL-methodology which takes advantage of changes in intensity of corresponding pixels in consecutive NIRS-IVUS frames and consists of a network model designed in a bidirectional gated-recurrent-unit (Bi-GRU) structure was trained to detect end-diastolic frames. The efficacy of the DL-methodology in identifying end-diastolic frames was compared with two expert analysts and a conventional image-based (CIB)-methodology that relies on detecting vessel movement to estimate phases of the cardiac cycle. A window of ± 100 ms from the ECG estimations was used to define accurate end-diastolic frames detection. The ECG-signal identified 3,167 end-diastolic frames. The mean difference between DL and ECG estimations was 3 ± 112 ms while the mean differences between the 1st-analyst and ECG, 2nd-analyst and ECG and CIB-methodology and ECG were 86 ± 192 ms, 78 ± 183 ms and 59 ± 207 ms, respectively. The DL-methodology was able to accurately detect 80.4%, while the two analysts and the CIB-methodology detected 39.0%, 43.4% and 42.8% of end-diastolic frames, respectively (P < 0.05). The DL-methodology can identify NIRS-IVUS end-diastolic frames accurately and should be preferred over expert analysts and CIB-methodologies, which have limited efficacy.

**Supplementary Information:**

The online version contains supplementary material available at 10.1007/s10554-021-02162-x.

## Introduction

Intravascular ultrasound (IVUS) is the preferred intravascular imaging modality for the evaluation of coronary plaque burden and the efficacy of emerging therapies targeting plaque evolution [1]. Grayscale-IVUS analysis in intravascular imaging studies is usually performed at 1-mm intervals ignoring the phase of the cardiac cycle [2], even though this approach neglects cyclical changes in luminal dimensions and the longitudinal motion of the IVUS catheter within the vessel, which can affect accurate quantification of atheroma [3–5]. It has been suggested that gated-IVUS analysis may be more accurate and reproducible [6, 7]; however, commercial catheters in contemporary use do not incorporate an electrocardiographic (ECG)-signal to allow the end-diastolic frame to be discerned. Whilst hardware for gated-IVUS acquisition has been developed, it has not found widespread use partly due to the increased time required for image acquisition [8]. Moreover, the automated methodologies developed to retrospectively-gate IVUS images, taking advantage of the relative movement of the lumen with regards to the IVUS catheter, have failed to dominate in research as most of them have not been robustly validated against ECG estimations as reference standard or they have not been incorporated in user-friendly software [9–17]. Over recent years, deep learning (DL) algorithms have gained interest as potential solutions for the rapid and accurate analysis of large datasets in cardiac imaging [18]. These approaches rely on the use of a pre-defined reference standard to train algorithms that can detect features on a training dataset and then apply these algorithms on new data. Despite the potential value of this approach, its application in intravascular imaging has not been fully explored yet [19–24].

It is hypothesised that a DL-based methodology will be able to detect end-diastolic frames in IVUS datasets and may be superior to human experts and previous automated methodologies. The aims of this study are: (a) to develop and train a novel DL-based methodology for automated detection of end-diastolic frames in IVUS sequences, (b) examine the reproducibility of expert analysts in detecting end-diastolic IVUS frames and (c) validate the performance of DL, the expert analysts and an established, conventional image-based (CIB)-methodology in detecting end-diastolic frames in patients with co-registered IVUS images and ECG-recordings.

## Methods

### Study population

Twenty coronary arteries from six consecutive patients in sinus rhythm who were recruited to the “Evaluation of the efficacy of computed tomographic coronary angiography (CTCA) in assessing coronary artery morphology and physiology” study (NCT03556644), were included in the present analysis. The rationale, study design, and inclusion and exclusion criteria have been presented in detail elsewhere [25]. Briefly, patients with stable angina and obstructive coronary disease on coronary angiography referred to Barts Heart Centre for invasive assessment were considered eligible for participation. All patients underwent CTCA followed by coronary catheterisation and 3-vessel near-infrared spectroscopy (NIRS)-IVUS imaging. Only native vessels were included in the study. The study protocol complied with the Declaration of Helsinki and was approved by the local research ethics committee. All recruited patients gave written informed consent.

### Intravascular ultrasound analysis and ECG co-registration

NIRS-IVUS imaging was performed using the 50 MHz Dualpro system (Infraredx, Burlington, Massachusetts, United States) according to a standardised protocol. After administration of 400mcg of nitrates the NIRS-IVUS probe was advanced to the distal part of the vessel and an angiographic projection was obtained under contrast agent injection to identify the location of the probe. The probe was pulled back at a constant speed of 0.5 mm/s using an automated pullback device (frame-rate: 30 frames per second (fps)). A concurrent ECG tracing was recorded at the time of pullback. The NIRS-IVUS data and the ECG tracing were imported in a viewer-mixer that enabled simultaneous visualisation of the two recordings; the displays of the viewer-mixer were recorded at 120fps and digital video files were transferred to an offline workstation for further analysis.

### Manual end-diastolic frame detection

Analysis was performed by two well-trained analysts (RB and YK) who have segmented > 375,000 NIRS-IVUS frames. The two analysts reviewed the digital-video files that simultaneously portrayed the NIRS-IVUS pullback and the ECG tracing and identified each NIRS-IVUS frame that corresponded to the peak of the R-wave as an end-diastolic frame. These ECG estimations were checked by a third analyst (CVB); any conflicts were resolved by consensus.

Two months after this process the two analysts reviewed the original NIRS-IVUS sequences blindly to the ECG and manually identified the end-diastolic frames; in this analysis, it was assumed that the NIRS-IVUS frame with the minimal lumen motion before the largest movement of the lumen in relation to the NIRS-IVUS catheter corresponded to the end-diastolic frame. The 1st-analyst performed the analysis twice (3 weeks apart) to report intra-observer variability while the estimations of the 2nd-analyst were used to report inter-observer variability.

To explore the effect of frame rate on the estimations of the two analysts, a second analysis was performed in NIRS-IVUS sequences with a frame rate of 15fps; these data were obtained after excluding 1 of every 2 frames from each sequence. Again, intra- and inter-observer variability were reported and their estimations compared to ECG estimations, which were calculated by halving the frame number of the initial ECG estimations.

### Conventional image-based gating algorithm

CIB-gating was performed in the NIRS-IVUS data using a special version of the QCU-CMS software (version 4.69, Leiden University Medical Center, Leiden, The Netherlands) by implementing an established solution, the only one that has previously been incorporated in a user-friendly software package for IVUS analysis [10]. This approach is further described in the Data Supplement. The CIB algorithm processed the NIRS-IVUS sequences twice and its intra-observer variability was reported.

### Deep learning methodology

**Fig. 1:**
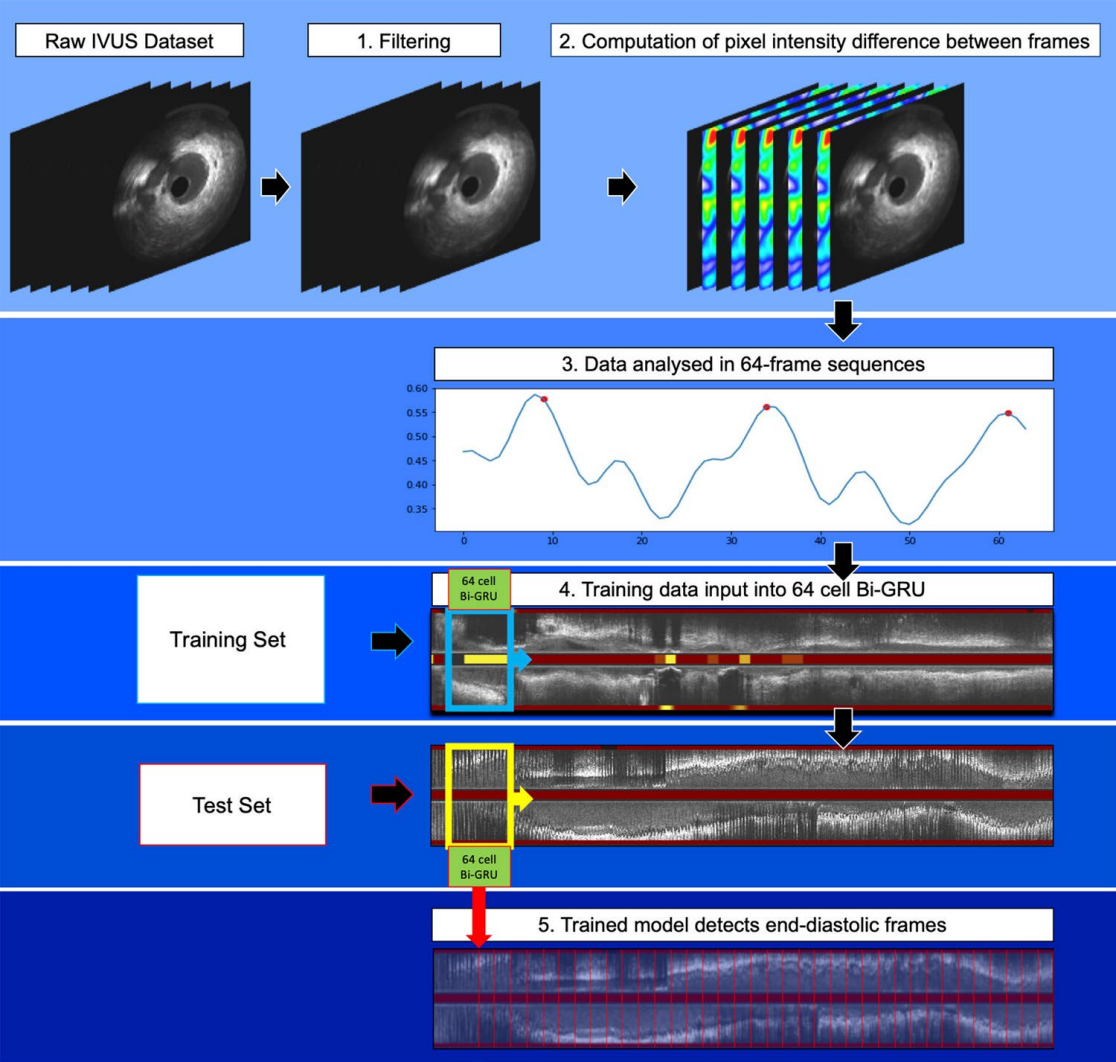
Schematic representation of the DL-methodology introduced for accurate detection of end-diastolic frames: (1) implementation of a Median filter to reduce noise in NIRS-IVUS images, (2) estimation of the absolute difference of grayscale-intensity of corresponding pixels identified in sequential frames, (3) schematic representation of the sum of the pixel differences that indicates the relative movement of the lumen with regards to the NIRS-IVUS probe; a segment of 64 frames is created that incorporates at least one cardiac cycle and sweeps the NIRS-IVUS sequence, (4) these data are used as a training set and entered into a 64-cell Bi-GRU (bidirectional-gated recurrent unit) neural network, (5) the trained network is used to process 64-frame segments generated in the test set and identify in this set the end-diastolic frames

We propose a novel DL-methodology for identifying end-diastolic frames in NIRS-IVUS sequences. The methodology consists of several steps (Fig. 1). First, a median filter was applied to reduce the impact of noise on the overall changes in the pixel intensity between consecutive NIRS-IVUS frames. Then, the absolute pixel-intensity difference between corresponding pixels in the entire image in consecutive NIRS-IVUS frames was calculated and these differences were summed up across the entire pullback. The obtained values indicate the relative motion of the vessel with regards to the NIRS-IVUS probe; this data was further smoothened by the Hanning smoothing algorithm applied on a 13-frame sliding window. The aim was to reduce the impact of noise on the frame-to-frame change data which forms the foundation for the subsequent vessel motion feature extraction process.

This sequence was processed by the DL-methodology which consists of a network model designed in a bidirectional gated-recurrent-unit (Bi-GRU) structure (Supplementary Fig. 1), capable of analysing data sequentially in both temporal directions, taking advantage of patterns extracted from frames before and after the end-diastolic frame [26]. This approach was found to be superior to a long short-term memory network model which was also tested as a potential solution. It is necessary to input a complete cardiac cycle into the network to identify the end-diastolic frame; however, an extra cardiac cycle can help the network better identify the motion of the vessel and localise the frame of interest. Based on these considerations, a meaningful sequence-length for training the DL-algorithm was assumed to contain at least two full cardiac cycles. The majority of end-diastolic frame intervals in the training dataset were between 19 and 30 frames, therefore a segment length of 64 frames was used. That segment started from the beginning of the smoothened sequence and advanced frame-by-frame until this swept the entire studied vessel. In this way, for every NIRS-IVUS sequence n-63 segments of 64 frames were generated where n is the number of NIRS-IVUS frames. These segments were regarded as the basic units for training the DL model; training was performed separately in the left anterior descending (LAD), left circumflex (LCx) and right coronary artery (RCA) using the ECG-defined end-diastolic frames to generate models for each vessel type.

The choice of loss function used for the Bi-GRU model was mean square error and learning rates in all experiments were set to $1{\mathrm{e}}^{-3}$. The maximum training epoch was 50 with batch size set to 1 during all experiments. As the model is ready-trained using the data without any manual fine tuning of hyperparameters, no validation set was required in this experiment.

Testing of the DL models was performed using the leave-one-out cross-validation approach—all pullbacks performed in the LAD, or the LCx, or the RCA were used for training apart from one vessel from each vessel type (LAD/LCx/RCA) that was used for testing. This process was repeated sequentially leaving a different vessel out from the training set until all vessels were used for testing. Similar to the training set, a 64-frame segment was generated that swept the entire NIRS-IVUS sequence. For each segment, the probability of the 32nd frame to be the end-diastolic frame was calculated and the frames with the highest probability amongst neighbouring frames were selected and constituted the network output. The DL-methodology analysed the NIRS-IVUS images twice and its intra-observer variability was reported.

### Statistical methods

**Fig. 2:**
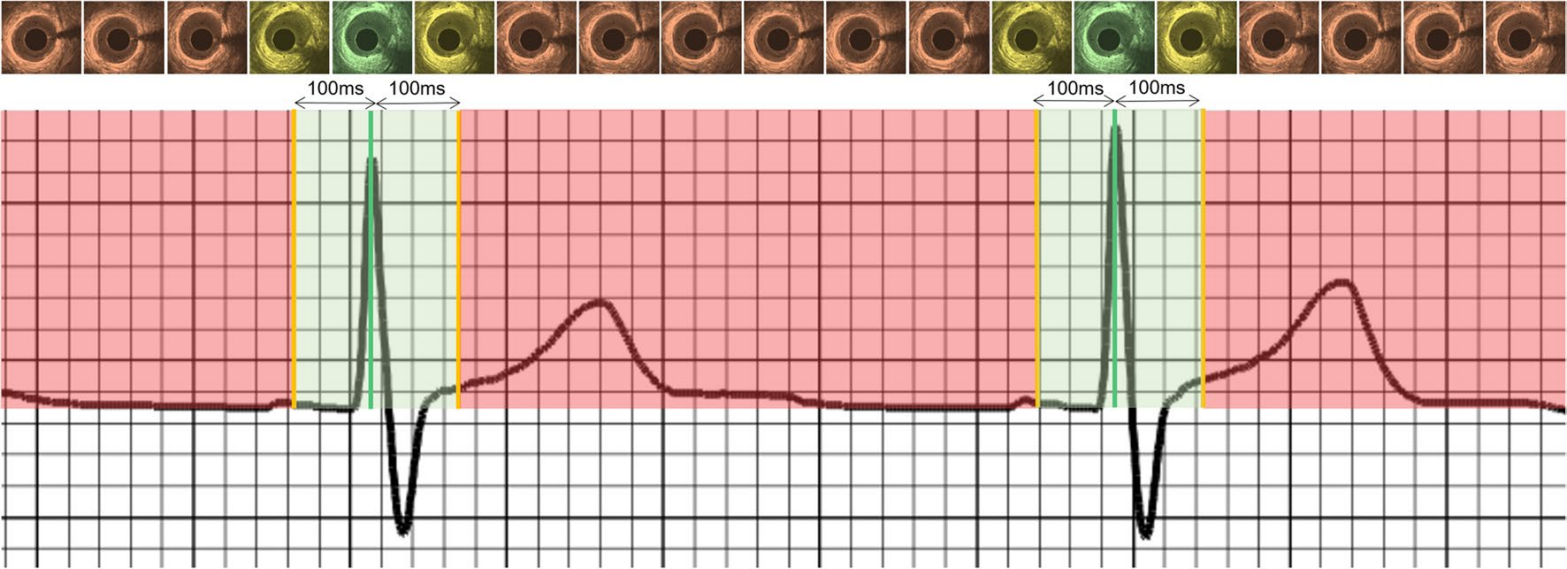
ECG recording with the corresponding NIRS-IVUS frames acquired at different phases of the cardiac cycle. The IVUS frame corresponding to the peak of the R-wave was defined by the ECG as the end-diastolic frame (green line). The green shaded area represents a period within ± 100 ms of the peak of R-wave. Frames detected by the analysts or the CIB- or DL-methodologies that fell in the green shaded area were classified as correctly detected end-diastolic frames. It is apparent in the NIRS-IVUS frames above the ECG that there is no motion of the lumen with regards to the NIRS-IVUS probe in frames that correspond to the end-diastolic period (shown with a green or a yellow colour); conversely the relative movement of the lumen with regards to the NIRS-IVUS probe is increased in the frames that do not correspond to the end-diastolic period and are portrayed with an orange colour

Numerical variables are presented as mean ± standard deviation (SD) while categorical variables are reported as absolute values and percentages. Comparison between categorical variables was performed using the Chi-square test. Bland–Altman analysis was used to assess the intra- and inter-observer variability of the analysts and compare their estimations and the estimations of the CIB- and DL-methodology against the end-diastolic frames identified by the ECG. The root mean square error (RMSE) between the estimations of the analysts, the CIB, the DL-methodology and the ECG were computed and compared. Considering that the temporal resolution of NIRS-IVUS imaging is 33.3 ms (30fps) we used a fixed cut-off range of ± 100 ms from the peak of R-wave on ECG to define correct end-diastolic frame detection (Fig. 2). In the absence of “true/false negatives” we defined the accuracy of each of the above approaches as the percentage of the correct end-diastolic frames from the end-diastolic frame estimations corresponding with a cardiac cycle. A P-value of < 0.05 was taken to be statistically significant. Statistical analyses were performed using SPSS for Mac version 23 (IBM, Armonk, New York).

## Results

### Studied patients

The baseline characteristics of the included patients are shown in Table 1. NIRS-IVUS imaging was performed in 20 vessels [6 LAD, 9 LCx (3 performed in the main vessel and 6 in major obtuse marginals) and 5 RCA]; the total number of acquired frames was 92,526, of which 3271 frames were classified as end-diastolic frames by ECG-signal. From these, we excluded 104 frames due to image artefacts obscuring detection of the peak of R-wave or identification of frame number. Therefore, in total 3167 frames were included in the final analysis.

**Table 1:** Baseline demographics of the studied patients

	All patients (N = 6)
Age (years)	61.7 ± 10.3
Gender (male)	5 (83.3%)
Current smoker	0 (0%)
Family history of CAD	4 (66.7%)
Co-morbidities	
Diabetes mellitus	3 (50.0%)
Hypertension	4 (66.7)
Hypercholesterolemia	4 (66.7)
Renal failure*	0 (0%)
Anemia	0 (0%)
Previous PCI	2 (33.3%)
LV function**	
Normal LV function	5 (83.3%)
Impaired LV function	1 (16.7%)
Studied vessels	(n = 20)
Left anterior descending artery	6 (30.0%)
Number of frames	32,171
Number of end-diastolic frames	1219
Left circumflex artery	9 (45.0%)
Number of frames	31,306
Number of end-diastolic frames	1070
Right coronary artery	5 (25.0%)
Number of frames	29,049
Number of end-diastolic frames	1008
Medications at discharge	
Aspirin	6 (100.0%)
Thienopyridines	6 (100.0%)
Beta-blocker	2 (33.3%)
Statin	6 (100.0%)

*CAD* coronary artery disease, *LV* left ventricle, *PCI* percutaneous coronary intervention

*Renal failure was defined as an estimated glomerular filtration rate of < 60 ml/min/1.73 m^2^

**Normal LV function was defined as LV ejection fraction ≥ 50%; impaired LV function was defined as LV ejection fraction > 30% and < 50%

### End-diastolic frame detection in NIRS-IVUS sequences with a frame-rate of 30fps

The representative analysis time for an analyst identifying end-diastolic frames manually for a representative 5000-frame vessel was approximately 15 min. Automated methodologies were significantly quicker: the CIB-methodology required 135 s while the DL-methodology required 15 s for the same number of frames to be analysed.

The 1st-analyst identified 3144 end-diastolic frames; 3034 estimations corresponded with the ECG-defined cardiac cycle; 133 estimations could not be matched with the ECG estimations as there were other annotations closer to the peak of R-wave, while in 110 cases the analyst failed to identify an end-diastolic frame for a cardiac cycle. Similar results were reported when analysis was repeated by the 1st-analyst or when end-diastolic frame detection was performed by the 2nd-analyst (Table 2). There was no difference between the analysts and the CIB-methodology in the incidence of frames that were matched with the ECG estimations, of frames that could not be matched to ECG estimations and to missed end-diastolic frames for a cardiac cycle (P = 0.182). Conversely, the DL-methodology missed cardiac cycles more frequently than the analysts or the CIB-methodology (P ≤ 0.010). Vessel-level analysis demonstrated that this difference was mainly due to a larger number of missed end-diastolic frames in the LAD. In the LCx, the analysts were more likely to detect additional end-diastolic frames not corresponding to ECG estimations than the CIB- and DL-methodologies. Finally, in the RCA, the CIB- and DL-methodologies were found to more frequently identify extra end-diastolic frames not detected on ECG than the two analysts.

**Table 2:** Vessel-level analysis of end-diastolic frame estimations of the 1st- and 2nd-analyst and of the CIB- and DL-methodologies in the NIRS-IVUS sequences acquired at 30fps

Studied vessel	Segmentation approach	End-diastolic frames corresponding to ECG estimations	End-diastolic frames not corresponding to ECG estimations	Missed cardiac cycles	P
All studied vessels	1st-analyst 1st estimation	3034 (92.6%)	110 (3.4%)	133 (4.1%)	
	1st-analyst 2nd estimation	3014 (93.1%)	72 (2.2%)	153 (4.7%)	
	2nd-analyst	3069 (93.5%)	117 (3.6%)	98 (3.0%)	< 0.001
	CIB-methodology	3052 (93.5%)	96 (2.9%)	115 (3.5%)	
	DL-methodology	2987 (91.6%)	95 (2.9%)	180 (5.5%)	
Left anterior descending artery	1st-analyst 1st estimation	1141 (94.7%)	23 (1.9%)	42 (3.4%)	
	1st-analyst 2nd estimation	1136 (94.4%)	22 (1.8%)	46 (3.8%)	
	2nd-analyst	1140 (94.7%)	22 (1.8%)	42 (3.5%)	< 0.001
	CIB-methodology	1119 (93.9%)	10 (0.8%)	63 (5.3%)	
	DL-methodology	1062 (88.8%)	14 (1.2%)	120 (10.0%)	
Left circumflex artery	1st-analyst 1st estimation	976 (92.8%)	41 (3.9%)	35 (3.3%)	
	1st-analyst 2nd estimation	968 (94.3%)	16 (1.6%)	43 (4.2%)	
	2nd-analyst	976 (92.6%)	43 (4.1%)	35 (3.3%)	< 0.001
	CIB-methodology	972 (94.0%)	23 (2.2%)	39 (3.8%)	
	DL-methodology	971 (94.5%)	16 (1.6%)	40 (3.9%)	
Right coronary artery	1st-analyst 1st estimation	917 (89.9%)	46 (4.5%)	57 (5.6%)	
	1st-analyst 2nd estimation	910 (90.3%)	34 (3.4%)	64 (6.3%)	
	2nd-analyst	953 (92.9%)	52 (5.1%)	21 (2.0%)	< 0.001
	CIB-methodology	961 (92.7%)	63 (6.1%)	13 (1.3%)	
	DL-methodology	954 (91.8%)	65 (6.3%)	20 (1.9%)	

*CIB* conventional image-based, *DL* deep learning, *ECG* electrocardiogram

**Fig. 3:**
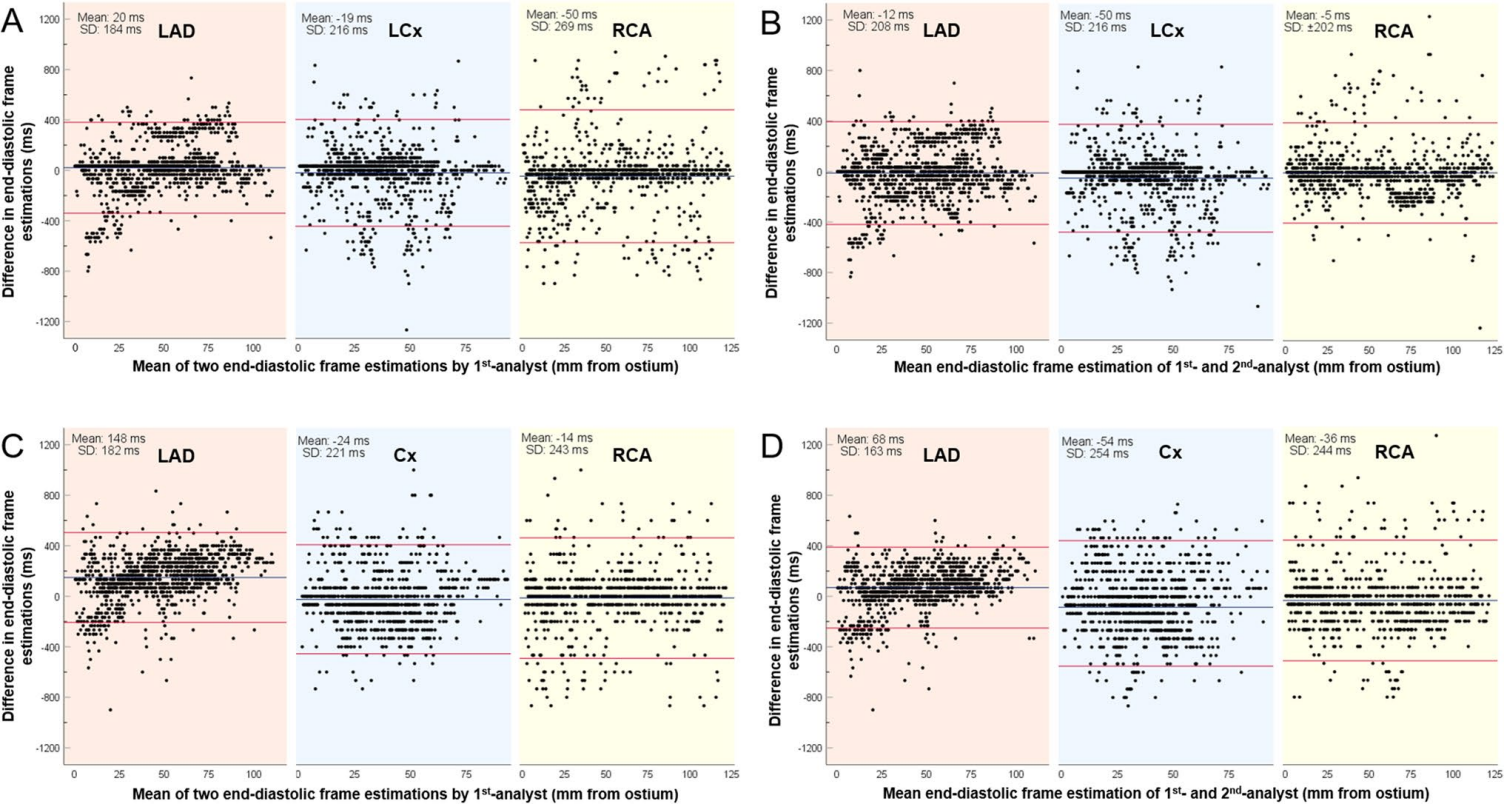
Bland–Altman analyses between the first and second set of estimations of the 1st-analyst at the NIRS-IVUS sequences acquired at 30fps (**a**) and 15fps (**b**) and between the estimations of the 1st- and 2nd-analyst in the same data set (**c** at 30fps; **d** at 15fps). Results are shown for the left anterior descending (LAD), left circumflex (LCx), and right coronary artery (RCA). The blue line represents the mean difference and the red lines correspond to the limits of agreement i.e., ± 1.96 standard deviation (SD)

Bland–Altman analysis demonstrated an intra- and inter-observer variability for the detected end-diastolic frames in all the studied vessels of 13 ± 224 ms and 23 ± 227 ms, respectively. This was in contrast to the CIB- and DL-methodologies which were perfectly reproducible, identifying exactly the same end-diastolic frames in repeated analyses. The vessel type did not impact the intra- or inter-observer agreement (Fig. 3).

**Fig. 4:**
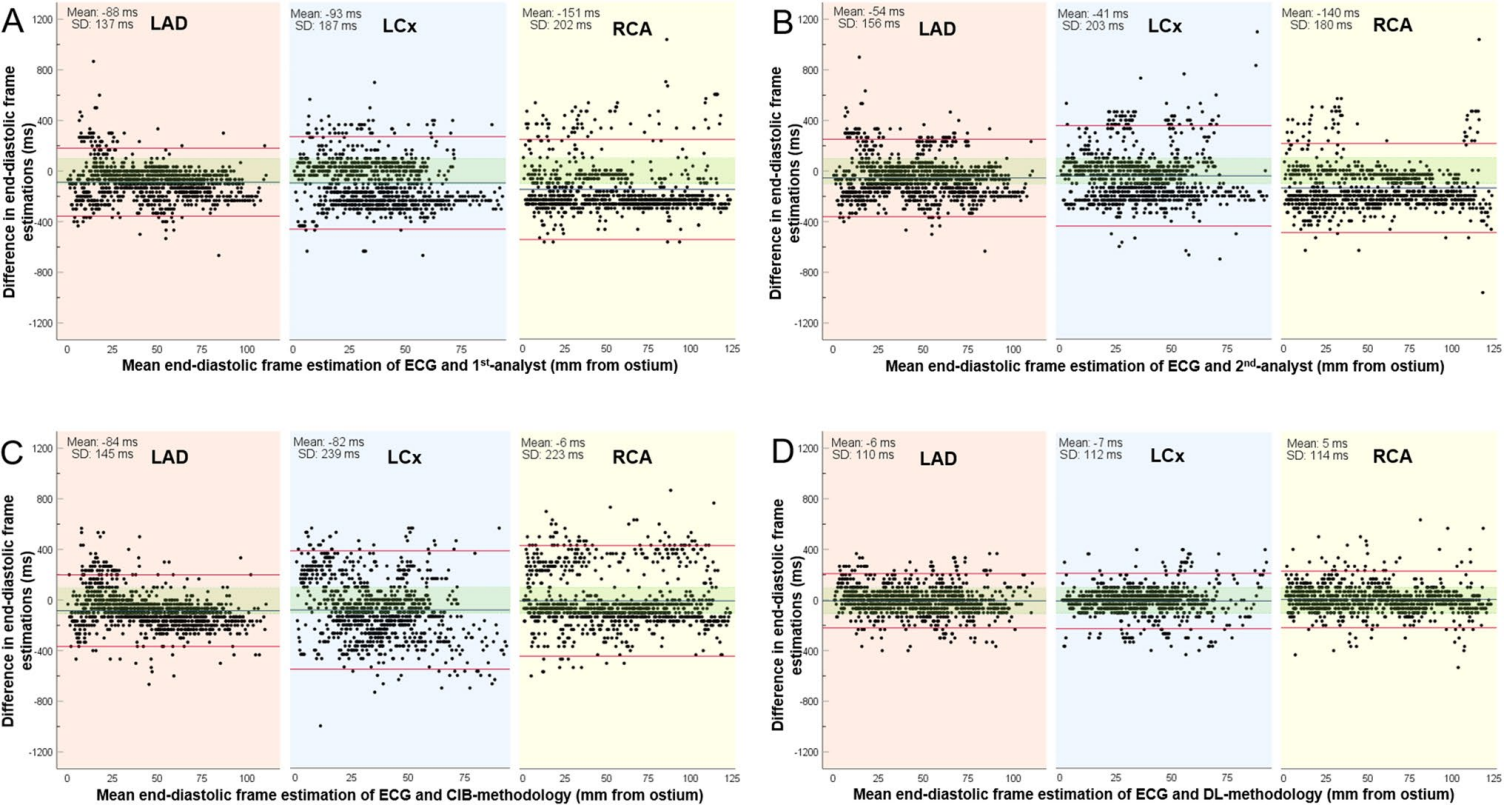
Bland–Altman analyses in the NIRS-IVUS sequences acquired at 30fps for the estimations of the ECG and the 1st-analyst (**a**), of the ECG and the 2nd-analyst (**b**), of the ECG and the CIB-methodology (**c**) and of the ECG and the DL-methodology (**d**). Results are shown for the left anterior descending (LAD), left circumflex (LCx), and right coronary artery (RCA). The blue line represents the mean difference and the red lines correspond to the limits of agreement, i.e. ± 1.96 standard deviation (SD). The green shaded area denotes estimations falling within ± 100 ms from the ECG estimations

Figure 4 illustrates the Bland–Altman analyses between ECG estimations and the estimations of the 1st-analyst, 2nd-analyst, CIB- and DL-methodologies. The differences between the estimations of the analysts and ECG or between the CIB-methodology and ECG were similar (86 ± 192 ms for the 1st-analyst, 78 ± 183 ms for the 2nd-analyst, 59 ± 207 ms for the CIB-methodology); conversely the DL-methodology was more accurate, with a smaller mean difference between its estimations and the ECG (3 ± 112 ms). These findings were consistent in all three coronary arteries. RMSE analysis also confirmed the superiority of DL-methodology (Table 3).

**Table 3:** Root mean square errors of the end-diastolic frame estimations of the analysts of the CIB- and DL-methodologies compared to ECG estimations

	1st-analyst	2nd-analyst	CIB-methodology	DL-methodology	P
Analysis at 30fps					
All studied vessels (ms)	194	184	199	103	< 0.001
Left anterior descending artery (ms)	154	156	157	100	< 0.001
Left circumflex artery (ms)	193	188	234	104	< 0.001
Right coronary artery (ms)	228	207	203	104	< 0.001
Analysis at 15fps					
All studied vessels (ms)	204	177	207	108	< 0.001
Left anterior descending artery (ms)	188	154	167	105	< 0.001
Left circumflex artery (ms)	175	179	240	108	< 0.001
Right coronary artery (ms)	245	198	212	112	< 0.001

*CIB* conventional image-based, *DL* deep learning

**Fig. 5:**
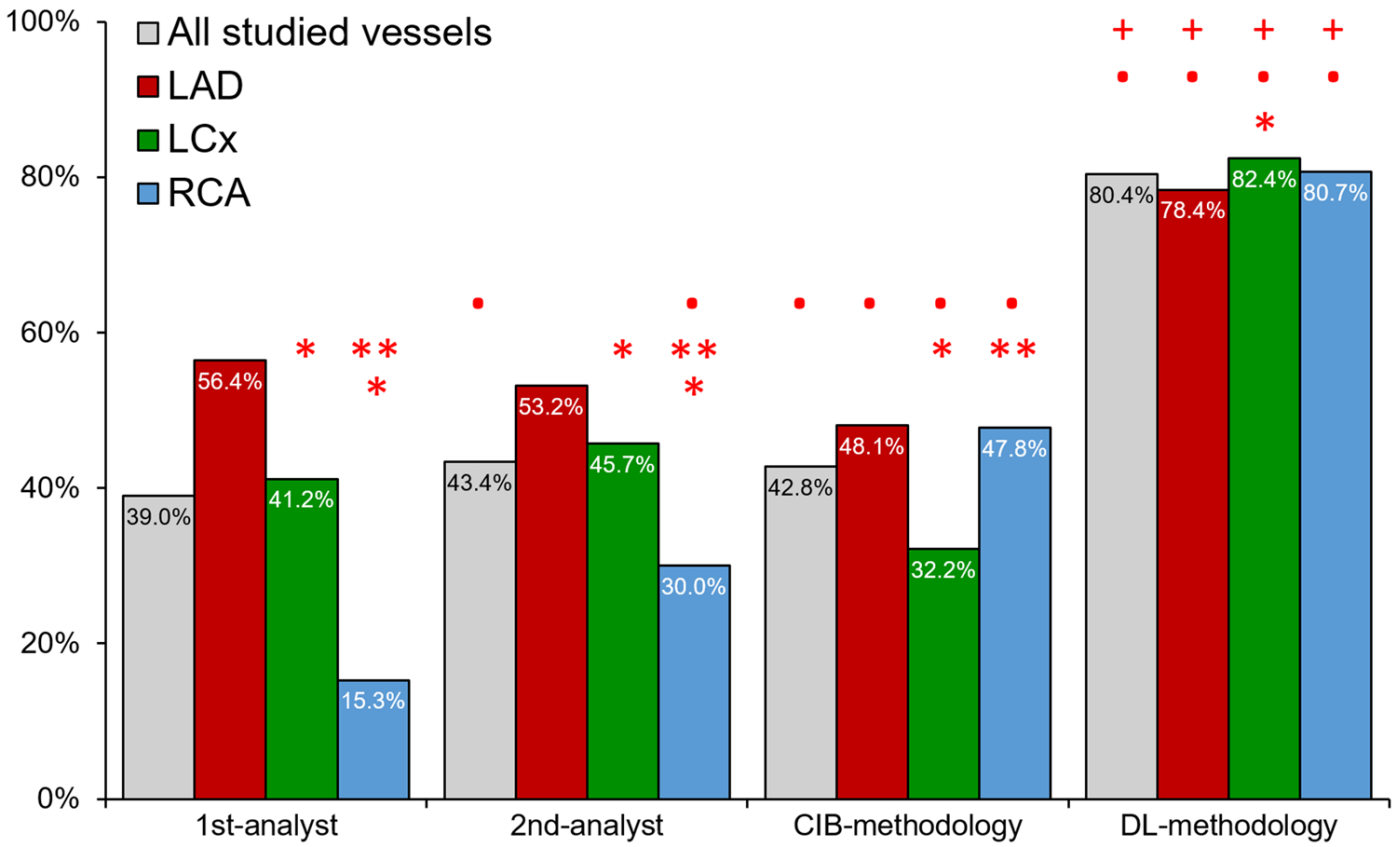
Accuracy of expert analysts, the CIB- and DL-methodology in the NIRS-IVUS sequences acquired at 30fps. (Red colour asterisk) Indicates statistically significant (P < 0.05) differences between the accuracy in left circumflex (LCx) or the right coronary artery (RCA) and the accuracy in the left anterior descending artery (LAD) within the same methodology. (Red colour double asterisk) Indicates statistically significant differences between the accuracy of the RCA and LCx within the same methodology. (Red colour bullet) Indicates statistically significant differences between the 1st-analyst and the 2nd-analyst, CIB- or the DL-methodology while red colour plus symbol indicates statistically significant difference between the CIB- and DL-methodology

**Fig. 6:**
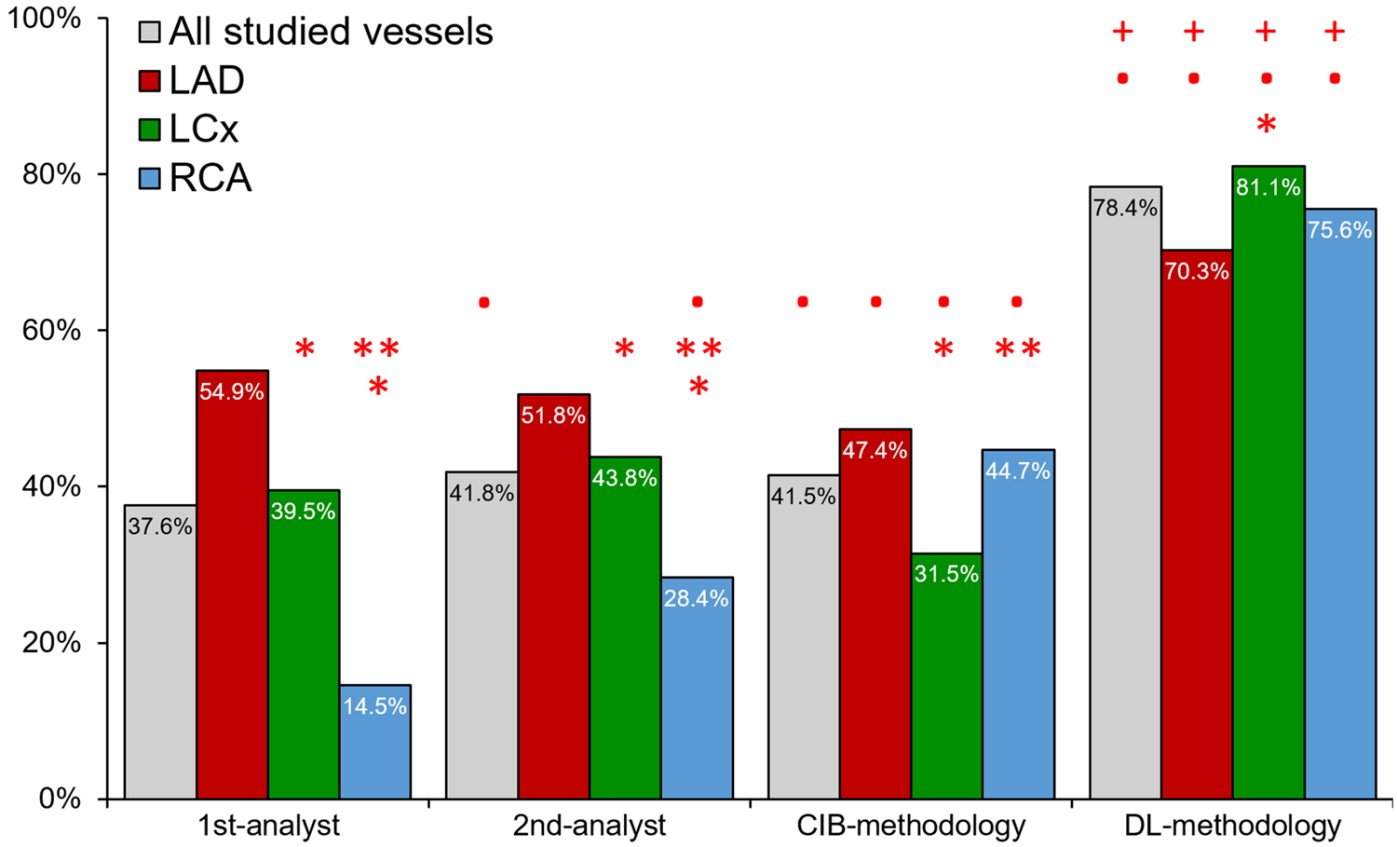
Accuracy of the expert analysts, the CIB- and DL-methodology in the NIRS-IVUS sequences acquired at 30fps. In this analysis accuracy was defined as the percentage of the correct estimations versus the number of all the estimated end-diastolic frames. (Red colour asterisk) Indicates statistically significant (P < 0.05) differences between the accuracy in left circumflex (LCx) or the right coronary artery (RCA) and the accuracy in the left anterior descending artery (LAD) within the same methodology. (Red colour double asterisk) Indicates statistically significant differences between the accuracy of the RCA and LCx within the same methodology. (Red colour bullet) Indicates statistically significant differences between the 1st-analyst and the 2nd-analyst, CIB- or the DL-methodology while red colour plus symbol indicates statistically significant difference between the CIB- and DL-methodology

Using a cut-off of ± 100 ms difference from ECG estimations to define correct end-diastolic frame detection, we found that the 1st-analyst correctly identified 1182 frames (39.0%), the 2nd-analyst 1333 (43.4%), the CIB-methodology 1306 (42.8%) and the DL-methodology 2401 (80.4%) frames (Fig. 5, P < 0.05). The accuracy of the analysts was significantly better for the LAD and LCx, compared to the RCA (P < 0.05), while the CIB-methodology was more accurate in the LAD and RCA compared to the LCx (P < 0.001). DL had excellent accuracy and was superior to the analysts and the CIB-methodology in all 3 coronary arteries and appeared to perform better in the LCx than the LAD. Results were not different when accuracy was defined as the ratio of correctly identified end-diastolic frames versus all frames estimated by each approach (Fig. 6).

The results were similar when we took into account patient heart rate variability and defined accurate end-diastolic frame detection as estimations falling within ± 10% of the R-R interval from the peak of R-wave. An accuracy of 68.0% was reported for the DL-methodology; the accuracy of the 1st-analyst was 35.6%, of the 2nd 29.7% and of the CIB-methodology 29.7%.

### End-diastolic frame detection in NIRS-IVUS sequences with a frame-rate of 15fps

The results from analysis at a frame-rate of 15fps are shown in Table 4. The CIB-methodology in this dataset detected more end-diastolic frames that did not correspond with ECG compared to the analysts or the DL-methodology. Vessel-level analysis indicated that this difference was driven by the performance of the CIB-methodology in the LCx and RCA. The DL-methodology performed similarly to the analysts in terms of detecting extra end-diastolic frames, but as before it missed more end-diastolic frames than the analysts or the CIB-methodology in the LAD.

**Table 4:** Vessel-level analysis of the end-diastolic frame estimations of the 1st- and 2nd-analyst and of the CIB- and DL-methodology in the NIRS-IVUS sequences acquired at 15fps

Studied vessel	Segmentation approach	End-diastolic frames corresponding to ECG estimations	End-diastolic frames not corresponding to ECG estimations	Missed cardiac cycles	P
All studied vessels	1st-analyst 1st estimation	2924 (90.4%)	68 (2.1%)	243 (7.5%)	
	1st-analyst 2nd estimation	2940 (90.9%)	66 (2.0%)	227 (7.0%)	
	2nd-analyst	3020 (93.2%)	72 (2.2%)	147 (4.5%)	< 0.001
	CIB-methodology	3036 (90.1%)	202 (6.0%)	131 (3.9%)	
	DL-methodology	2993 (93.4%)	39 (1.2%)	174 (5.4%)	
Left anterior descending artery	1st-analyst 1st estimation	1121 (93.5%)	17 (1.4%)	61 (5.1%)	
	1st-analyst 2nd estimation	1182 (100.0%)	0 (0.0%)	0 (0.0%)	
	2nd-analyst	1121 (93.3%)	19 (1.6%)	61 (5.1%)	< 0.001
	CIB-methodology	1111 (92.2%)	23 (1.9%)	71 (5.9%)	
	DL-methodology	1093 (91.6%)	11 (0.9%)	89 (7.5%)	
Left circumflex artery	1st-analyst 1st estimation	887 (86.7%)	12 (1.2%)	124 (12.1%)	
	1st-analyst 2nd estimation	949 (89.8%)	46 (4.4%)	62 (5.9%)	
	2nd-analyst	965 (93.2%)	24 (2.3%)	46 (4.4%)	< 0.001
	CIB-methodology	973 (90.4%)	65 (6.0%)	38 (3.5%)	
	DL-methodology	958 (94.0%)	8 (0.8%)	53 (5.2%)	
Right coronary artery	1st-analyst 1st estimation	916 (90.4%)	39 (3.8%)	58 (5.7%)	
	1st-analyst 2nd estimation	809 (81.4%)	20 (2.0%)	165 (16.6%)	
	2nd-analyst	934 (93.1%)	29 (2.9%)	40 (4.0%)	< 0.001
	CIB-methodology	952 (87.5%)	114 (10.5%)	28 (2.0%)	
	DL-methodology	942 (94.8%)	20 (2.0%)	32 (3.2%)	

*CIB* conventional image-based, *DL* deep learning, *ECG* electrocardiogram

Similar to the 30fps analyses, the intra- (26 ± 240 ms for all vessels) and inter-observer (26 ± 221 ms for all vessels) variability was higher for the estimations of the analysts when compared to the CIB- and DL-methodologies that were again perfectly reproducible (Fig. 3).

Bland–Altman analyses of frames identified by the ECG, 1st-analyst, 2nd-analyst, CIB- and DL-methodologies are shown in the Supplementary Fig. 2. The agreement between DL and ECG estimations (3 ± 117 ms) was higher than the agreement between ECG and 1st-analyst (76 ± 315 ms), 2nd-analyst (46 ± 186 ms) or CIB-methodology (13 ± 223 ms). These findings were consistent in all three coronary arteries. RMSE analysis again confirmed the superiority of the DL-methodology (Table 3). The DL-methodology had a significantly higher accuracy than the analysts or the CIB approach in detecting the correct end-diastolic frame in all 3 coronary arteries (Supplementary Fig. 3).

## Discussion

In this study, we have introduced, trained and tested for the first time a novel DL-methodology for detecting end-diastolic frames in NIRS-IVUS sequences using ECG-defined estimations as the reference standard. The novelty of the proposed framework relies on the use of a Bi-GRU network, for learning and processing in a bidirectional fashion the temporal information in IVUS sequences to detect end-diastolic frames. We found that, (1) expert analysts have limited accuracy and reproducibility in identifying end-diastolic frames, (2) the CIB-methodology, while more reproducible, has similar accuracy to expert analysts, (3) the efficacy of the analysts and CIB-methodology varies dependent on vessel type and (4) the DL-methodology is superior to expert analysts and the CIB-methodology, and has an excellent accuracy in identifying end-diastolic frames in all three coronary arteries.

The merits of analysing IVUS images obtained at the same phase of the cardiac cycle to accurately quantify volumetric changes in atheroma burden in longitudinal IVUS studies are well recognised. Previous studies have demonstrated that the lumen area can vary significantly, by as much as 10% in disease-free segments, during the cardiac cycle [4, 5]. Moreover, reports have demonstrated that the longitudinal motion of the IVUS catheter in the vessel during a cardiac cycle can reach up to 5.5 mm [3, 27]. A simulation study conducted by de Winter et al. showed that these factors can impact the efficacy of non-gated IVUS analysis in detecting changes in plaque burden in longitudinal studies; in their analysis, to detect a 3% decrease in plaque burden at follow-up, gated-IVUS analysis would require 26 vessels, while non-gated IVUS 254 vessels [7]. These findings were not confirmed by a report which included 19 vessels which were assessed twice by gated and non-gated IVUS; in this study, the differences in lumen, external elastic membrane and plaque volume between the two IVUS examinations were numerically smaller when analysis was performed using an ECG-gated device, but not statistically significant from the differences reported in the non-gated IVUS analysis [28]. This may be explained by the fact that the study is lacking the power to demonstrate the superiority of the ECG-gated analysis.

Hardware designed to selectively acquire gated IVUS frames [8] and automated image-based methodologies [9–17] to estimate end-diastolic frames have failed to dominate in clinical research. The lack of commercial availability of ECG-gated hardware and the increased time required for their use has limited their utility, while most of the CIB-methodologies have not been validated robustly against ECG estimations or incorporated in user-friendly software.

In the present study, we validated the efficacies of a CIB-methodology and expert analysts in 3,167 end-diastolic frames from 20 vessels assessed by NIRS-IVUS imaging against an ECG-defined reference standard. We found that the analysts had high intra- and inter-observer variability in detecting end-diastolic frames and that there were significant differences between their estimations and the ECG and that their performance varied between vessels. Both analysts performed better in the LAD and worse in the RCA where there is a higher relative motion of the lumen with regards to the catheter. In our analysis, the CIB-methodology was perfectly reproducible but its performance in detecting end-diastolic frames was limited and not different from the experts’. Significant variations were noted in the performance of the CIB-methodology in different vessels, with better performance in the LAD compared to the LCx and RCA.

Conversely, the proposed DL-methodology trained on the reference standard, taking into account the different patterns of motion in different vessels, appeared superior to the analysts and CIB-methodology with excellent accuracy in detecting end-diastolic frames overall and was not affected by the type of vessel.

The results were not different when we took into account the variability in the R-R interval between patients and used a cut-off of ± 10% of the R-R to define accurate end-diastolic frame detection. DL was again superior to the analysts and the CIB-methodology but it had a smaller accuracy than the one reported with the fixed cut-off of ± 100 ms. This should be attributed to the fact that the average heart rate of the studied patients was 73 ± 18 bpm but also to the limited temporal resolution of NIRS-IVUS imaging (33.3 ms). For the latter reason we believe that the fixed cut-off may allow a more accurate estimation of the performance of the DL-methodology especially in patients that had an increased heart rate during pullback.

The fixed cut-off that we used in our study also allowed us to examine the performance of the DL-methodology in IVUS sequences with a frame-rate of 15fps. The analysts had poor reproducibility and, similar to the CIB-methodology, limited efficacy in detecting end-diastolic frames while the DL-methodology did not appear to be affected by the lower spatial resolution and had excellent performance. These findings highlight the potential value of DL in processing intravascular imaging data acquired at a higher pull-back speed, or a lower frame-rate such as the NIRS-IVUS images acquired by the MC-9 system which obtains images at 14fps or the upcoming IVUS-photoacoustic imaging catheters that are expected to acquire images at a lower frame-rate than conventional IVUS systems [29].

Another advantage of the developed DL-methodology is that it is rapid, enabling processing of large datasets within a few seconds: analysis of an IVUS sequence of 5000 frames takes less than 15 s in a computer with an Intel-i5 processor and 16 GB RAM. The proposed approach has also been incorporated in the user-friendly QCU-CMS software, widely used for the processing of intravascular imaging data and this is likely to facilitate its utility in research. Moreover, the DL-methodology is expected to have applications in future computational fluid dynamic (CFD) studies assessing the implications of the local hemodynamic forces on plaque evolution. Recently, convincing evidence has emerged highlighting the role of coronary blood flow patterns on plaque progression and destabilisation [30, 31], while reports have shown that multimodality intravascular imaging combined with CFD estimations enables more accurate assessment of plaque pathophysiology and progression [32]. However, accurate reconstruction of coronary anatomy requires fusion of angiography with intravascular images at the end-diastolic phase of the cardiac cycle. None of the recently developed high-resolution IVUS or hybrid-IVUS systems incorporate ECG-gated hardware or are able to visualise the ECG alongside the acquired IVUS images. The developed DL-methodology overcomes these limitations, enabling retrospective, fast and accurate identification of end-diastolic frames necessary for reliable reconstruction of vessel architecture.

### Limitations

The present study has several limitations that should be acknowledged. Firstly, the number of vessels included was small and thus we could not examine the implications of the erroneous estimations of the analysts and of the CIB-methodology on the quantification of the lumen and vessel volumes [6]. Secondly, there was a variability in the number of vessels between LAD, LCx and RCA groups; however, the number of end-diastolic frames in each group was relatively large (> 1000 frames/group, Table 1) and similar in the 3 vessel groups, while the non-end-diastolic frames add up to around 85,000; both end-diastolic frames and non-end-diastolic frames are considered in training to highlight the differences of positive and negative training samples. Using leave-one-out cross-validation scheme, the size of training set for each validation is approximately 25,000 frames. Therefore, we believe that the obtained data were sufficient to adequately train the DL-methodology [33].

All included patients were in sinus rhythm, with a heart rate between 45 and 85 beats-per-minute and had a relatively stable R-R interval; nevertheless, premature atrial or ventricular ectopics were often seen during the pull-back and were included in the analysis. Therefore, the developed DL-methodology should be used only in patients in sinus rhythm as it is uncertain how significant rhythm variability might affect its performance. Moreover, the present analysis excluded stented segments where the friction effect between the catheter and the protruded struts may affect the movement of the lumen with regards to the IVUS probe—further research is needed to examine the performance of the proposed methodology in such segments. Finally, this study did not examine the value of IVUS-gating in the accurate quantification of atheroma burden compared to non-gated IVUS; this should be tested in a large-scale appropriately sized study.

## Conclusion

In conclusion, the proposed DL-methodology is capable of detecting end-diastolic frames in 50 MHz NIRS-IVUS datasets of all three coronary arteries rapidly and reproducibly, with greater accuracy than both expert analysts and a CIB-methodology. The DL-methodology’s performance is unaffected by the rate at which IVUS images are acquired. These features render it a useful tool for the analysis of IVUS datasets in longitudinal studies and in studies that fuse IVUS and X-ray angiographic data to assess the implication of the haemodynamic forces on plaque evolution.

## Supplementary Information

Below is the link to the electronic supplementary material.

Supplementary Information 1 (DOCX 27125 kb)

## Data Availability

Custom code not publicly available.
