# Supplementary material for: A deep learning methodology for the automated detection of end-diastolic frames in intravascular ultrasound images
Source: Int J Cardiovasc Imaging. 2021 Feb 15;37(6):1825–37. doi: 10.1007/s10554-021-02162-x (PMC8255253; doi:10.1007/s10554-021-02162-x)
Supplement: Supplementary file 1 — Supplementary Information 1 (DOCX 27125 kb) [file 10554_2021_2162_MOESM1_ESM.docx]

**A deep learning methodology for the automated detection of end-diastolic frames in intravascular ultrasound images – data supplement**

Retesh Bajaj, MBBS, BSc,^1,2,#,*^ Xingru Huang, BEng,^3,#,*^ Yakup Kilic, MD,^1,*^ Ajay Jain, MD,^1,*^ Anantharaman Ramasamy, MBChB,^1,2,*^ Ryo Torii, MSc, PhD,^4,*^ James Moon, MD,^1,5,*^ Tat Koh, MBBS, MD,^1,*^ Tom Crake, MD,^1,*^ Maurizio K. Parker, BSc, MRes,^2,*^ Vincenzo Tufaro, MD,^1,2,*^ Patrick W. Serruys, MD, PhD,^6,*^ Francesca Pugliese, MD, PhD,^1,2,*^ Anthony Mathur, MD, PhD,^1,2,*^ Andreas Baumbach, MD, PhD,^1,2,*^ Jouke Dijkstra, PhD,^7,*^ Qianni Zhang, PhD,^3,*^ Christos V. Bourantas, MD, PhD^1,2,5,*^

^1^ Department of Cardiology, Barts Heart Centre, Barts Health NHS Trust, London, UK

^2^ Centre for Cardiovascular Medicine and Devices, William Harvey Research Institute, Queen Mary University of London, UK

^3^ School of Electronic Engineering and Computer Science, Queen Mary University of London, UK

^4^ Department of Mechanical Engineering, University College London, London, UK

^5^ Institute of Cardiovascular Sciences, University College London, London, UK

^6^ Faculty of Medicine, National Heart & Lung Institute, Imperial College London, UK

^7^ Department of Radiology, Division of Image Processing, Leiden University Medical Center, Leiden, The Netherlands

^#^ The 1^st^ and 2^nd^ author contributed equally to this work

*Statement of authorship: This author takes responsibility for all aspects of the reliability and freedom from bias of the data presented and their discussed interpretation.

**Short title****:** Deep learning for end-diastolic frame detection in IVUS

*Conventional image-based gating algorithm*

This relies on the detection of neighbouring frames where the lumen motion is minimal as this has been correlated with the end-diastolic frame in the ECG-signal [1]. The methodology consists of three steps:

1) Image pre-processing: this step is used to reduce the typical speckle noise and unwanted structures like stent struts and involves the application of a Gaussian filter to smooth the images and remove regions of high intensities such as stent struts, which are expected to have large influence in the absolute difference measurements.

2) Generation of a signal that indicates the motion between neighbouring frames: this step involves definition of a donut shape structure that is delineated by the outer border of the NIRS-IVUS catheter artefact region and the outer border of the field of view of the NIRS-IVUS image. In this structure the sum of the absolute difference of the intensity of corresponding pixels between two neighbouring frames is computed. The sum of the intensity difference can be plotted against the frame numbers and result in a diagram, which shows periodical minima similar to an ECG signal. From this signal, the frames with the minimal motions are determined.

3) Filtering of the acquired signal to remove extrema: in this step, the signal is analysed by autocorrelation to provide the average ECG frequency for each NIRS-IVUS pullback. Since there is a heart rate variability during the NIRS-IVUS pullback, we search for the end-diastolic frame at a frequency that can range from 0.75 to 1.5 times the average ECG frequency. Candidate end-diastolic frames at a frequency above or below that range are discarded.

**Figure legends**

**Supplementary figure 1.** A schematic representation of the bidirectional (Bi-GRU) neural network structure with length $n$. $\vec{h}_{1}$ and $h_{1}$ represents GRU cells towards the forward and backward directions; $x$ represents the input to the model, in this case a 64-frame segment. The final, trained softmax function acts as the final layer of the neural network-based classifier, receiving the feedback from all GRU cells and calculating the probability of the frame being the end-diastolic frame.

**Supplementary figure 2.** Bland-Altman analyses in the NIRS-IVUS sequences acquired at 15fps for the estimations of the ECG and the 1^st^-analyst (A), of the ECG and the 2^nd^-analyst (B) of the ECG and the CIB- (C) and of the ECG and the DL-methodology. Results are shown for the left anterior descending (LAD), left circumflex (LCx), and right coronary artery (RCA). The blue line represents the mean difference and the red lines correspond to the limits of agreement, i.e. ±1.96 standard deviation (SD). The green shaded area denotes estimations falling within ±100ms from the ECG estimations.

**Supplementary figure 3.** Accuracy of the expert analysts, the CIB- and DL-methodology in the NIRS-IVUS sequences acquired at 15fps. * indicates statistically significant (P<0.05) differences between the accuracy in left circumflex (LCx) or the right coronary artery (RCA) and the accuracy in the left anterior descending artery (LAD) within the same methodology. ** indicates statistically significant differences between the accuracy of the RCA and LCx within the same methodology. • indicates statistically significant differences between the 1^st^-analyst and the 2^nd^-analyst or the CIB- or the DL-methodology while + indicates statistically significant difference between the CIB- and DL-methodology.

**Supplementary Figure 1.**


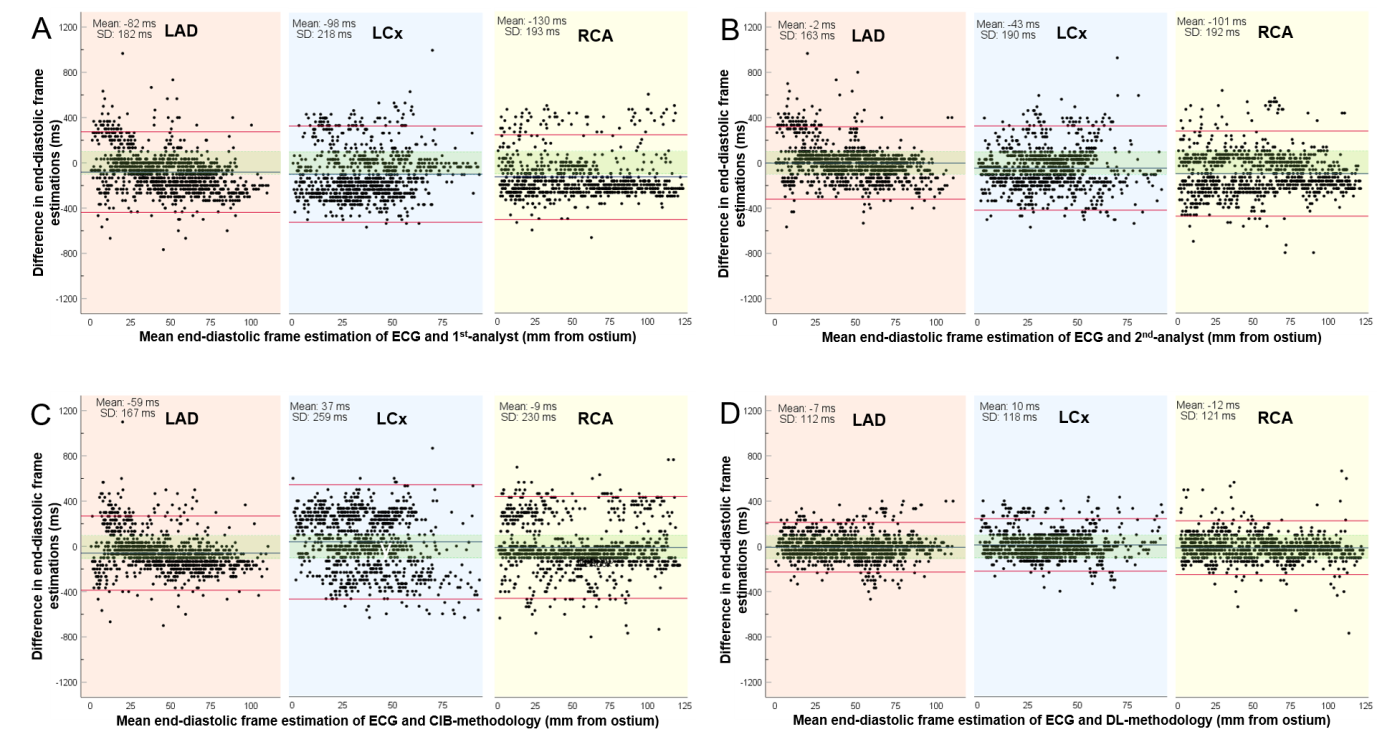


**Supplementary Figure 2.**


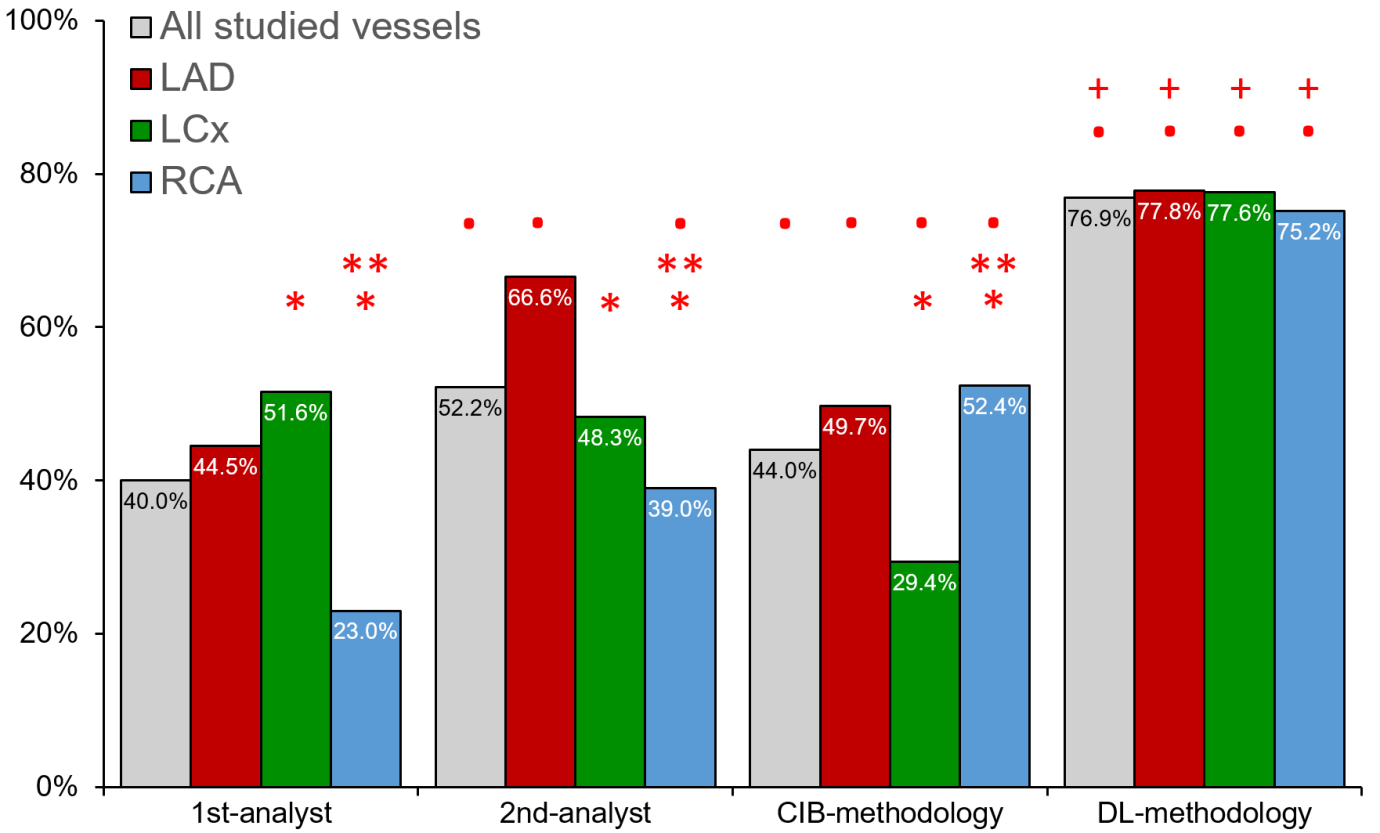


**Supplementary Figure 3**

**References**

1. de Winter SA, Hamers R, Degertekin M, et al (2003) A novel retrospective gating method for intracoronary ultrasound images based on image properties. In: Computers in Cardiology, 2003. IEEE, Thessaloniki Chalkidiki, Greece, pp 13–16
